# Supplementary figures and images for: RETINA: Reconstruction-based pre-trained enhanced TransUNet for electron microscopy segmentation on the CEM500K dataset
Source: PLoS Comput Biol. 2025 May 28;21(5):e1013115. doi: 10.1371/journal.pcbi.1013115 (PMC12143494; doi:10.1371/journal.pcbi.1013115)

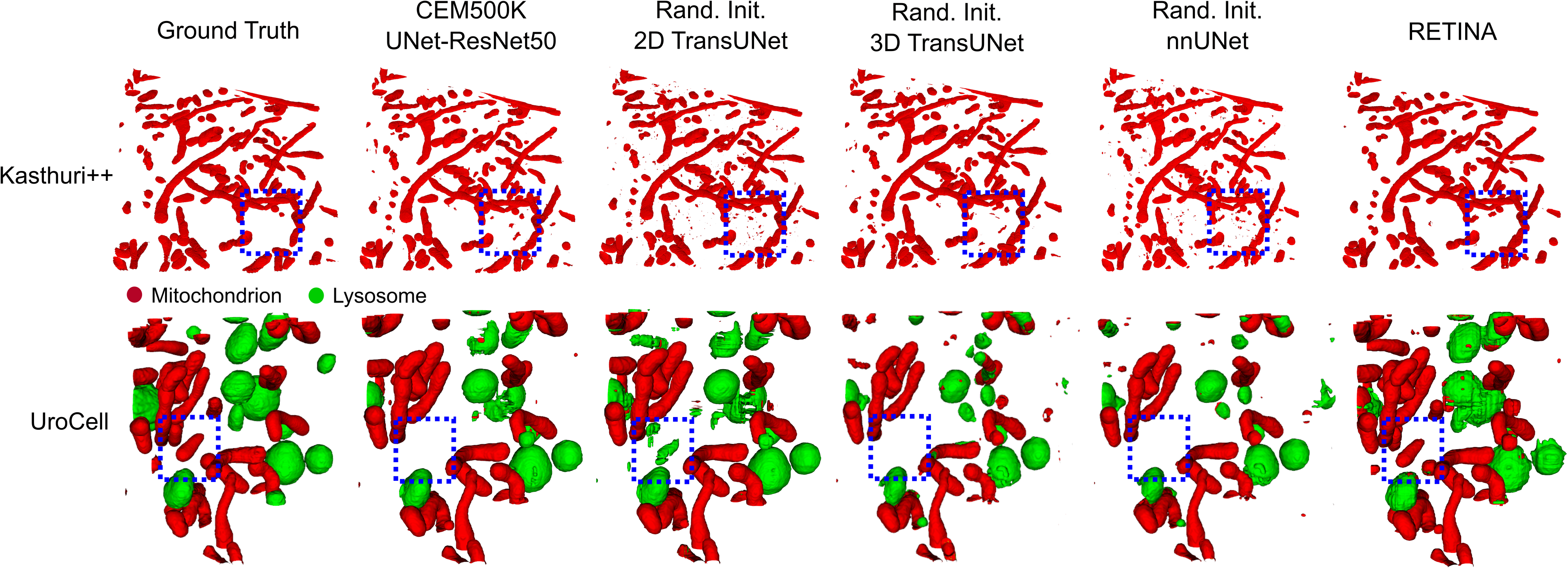

Supplement: S1 Fig — Labels for the Kasthuri++ and UroCell datasets are shown in the first and second rows, respectively. The dashed-line squares highlight regions where RETINA demonstrates improved segmentation accuracy. (TIFF) [file pcbi.1013115.s001.tiff]
