# Supplementary material for: RETINA: Reconstruction-based pre-trained enhanced TransUNet for electron microscopy segmentation on the CEM500K dataset
Source: PLoS Comput Biol. 2025 May 28;21(5):e1013115. doi: 10.1371/journal.pcbi.1013115 (PMC12143494; doi:10.1371/journal.pcbi.1013115)
Supplement: S1 Text — (PDF) [file pcbi.1013115.s002.pdf]

METHODS

RETINA Pre-training Methods

RETINA preprocesses each input 2D image with augmentation. Each input is duplicated once to form a pair: one is designated as ImageA and the other as ImageB. ImageB is saved as the original image and damaging augmentations are applied to ImageA. The augmentations are classified into two sets, implemented using the 'albumentations' Python package (Buslaev et al., 2020). The first set comprises 'HorizontalFlip', 'VerticalFlip', 'Rotate', and 'RandomResizedCrop', applied to both images in the pair. These don't damage any pixels. The second set includes 'RandomBrightnessContrast', 'GaussNoise', 'GaussianBlur', and 'CoarseDropout'. These damage pixels, so are applied only to ImageA, with ImageB serving as the reference for comparison. The parameters for each augmentation function are detailed in Table S1.

RETINA encodes ImageA using a combination of convolutional and Transformer layers, following the TransUNet architecture. Rather than employing a pure Transformer-based encoder, 2D convolutional layers are first applied to extract features from the input images. Patch embedding is then performed on the feature map extracted by the convolutional layers, where 1×1 patches are mapped into a latent embedding space using a trainable linear projection. To preserve spatial information, position embeddings are added to the patch embeddings, encoding positional relationships within the image. Following Transformer-based feature extraction, the resulting feature vector is represented as $z \in \mathbb{R}^{\frac{HW}{P^2} \times D}$, where H and W denote the image height and width, respectively, and each patch has a size of $P \times P$. D is the dimension of embedding space. To restore spatial structure, a reshaping step is applied, converting $\frac{HW}{P^2}$ back to $\frac{H}{P} \times \frac{W}{P}$, ensuring compatibility with the decoder input. To convert the embedding space back to the space of the input image, a 1×1 convolution is employed to reshape the feature representation to match the number of output classes. Finally, the feature map is upsampled to full resolution, producing an output of size $H \times W$, which aligns with the reconstructed image dimensions. See further details in (Chen et al., 2021).

Among the various TransUNet configurations, the ResNet50 and ViT-B/16 configuration are selected because ViT-B/16, when combined with ResNet50, has demonstrated excellent performance in previous studies (Chen et al., 2021, 2023). Additionally, ViT-B/16 offers a more compact structure compared to the L/16 variant, and we further optimized the model by reducing the number of parameters (Table S2) to balance high accuracy with a reduced training burden. The Transformer encoder consists of Multihead Self-Attention (MSA) layers and Multi-Layer Perceptron (MLP) layers (Dosovitskiy et al., 2020), with the output after the encoder given by:

$$z_\ell = \mathrm{MLP}(\mathrm{LN}(\mathrm{MSA}(\mathrm{LN}(z_{\ell-1}))) + z_{\ell-1}) + \mathrm{MSA}(\mathrm{LN}(z_{\ell-1})) + z_{\ell-1}, \tag{S1}$$

where LN() denotes the normalization operator, and z_ℓ is the encoded representation of the l-th layer. The architecture is illustrated in Figure 1a. Additionally, skip-connections are set to 3 to enable efficient feature aggregation at different resolution levels. After decoding the features obtained from the encoder, a 3x3 convolutional layer is applied to recover the decoded feature instead of the segmentation head used in the original TransUNet architecture. Finally, the MSE loss is calculated between the reconstructed output and ImageB. The overall parameter settings are detailed in Table S2. RETINA is implemented with PyTorch in a Python 3.10.2 environment and pre-trained for 200 epochs on Nvidia A100 GPUs on the Digital Research Alliance of Canada's Narval cluster. The training was distributed across 4 nodes, each with 4 GPUs, and completed in less than 2 days.

RETINA Fine-tuning Methods

Since the pre-trained layers are 2D-based, the fine-tuning architecture must also be compatible with 2D inputs. For 3D input images, the volume is first sliced into 2D images before being processed by the model. The same encoder and decoder parameter settings used during pre-training are retained for fine-tuning (Table S3). Pre-trained Transformer layer parameters are transferred to the fine-tuning Transformer layers and remain frozen during training. After decoding features from the latent space, a segmentation head—comprising a convolutional layer followed by bilinear upsampling—assigns a class label to each pixel, generating the final segmentation output. For multi-class segmentation tasks, the final loss minimized by RETINA is given by (Chen et al., 2021):

$$L = \lambda L_{\text{CE}} + (1-\lambda)L_{\text{D}} \tag{S2}$$

where λ is in the range [0,1], L_{CE} stands for cross-entropy loss, and L_{D} represents dice loss. For binary tasks, the loss is given by (Chen et al., 2021):

$$L = \lambda L_{\text{BCEL}} + (1-\lambda)L_{\text{BD}} \tag{S3}$$

$$L_{\text{BCEL}} = -\frac{1}{N}\sum_{i=1}^{N}\left[y_i \log(p_i) + (1-y_i)\log(1-p_i)\right] \tag{S4}$$

where L_{BD} is the binary dice loss, L_{BCEL} is the binary cross-entropy loss, N is the number of pixels, y_i is the true label for the ith pixel, and p_i is the predicted probability that the pixel belongs to the positive class. In both multi-class and binary tasks, λ is set to 0.5. Fine-tuning was implemented on Nvidia T4 GPUs on the Digital Research Alliance of Canada's Graham cluster, with multi-GPU computation deployed to reduce computation time.

Inference Methods

All benchmarks were inferred as 2D segmentation tasks, slice by slice. For UroCell, predictions were made on the xy, yz, and xz cross-sections due to its isotropic voxels, and the scores from each cross-section were averaged to obtain the final result. For anisotropic volumes, including the CREMI Synaptic Clefts, Guay, and Kasthuri++, they were predicted along a single direction. In the Perez dataset, since images were randomly sampled in 2D (Perez et al., 2014), the inference was simply applied in 2D. Evaluation followed the guidelines outlined in the CEM500K publication (Conrad and Narayan, 2021). For the Guay dataset, the first volume was used for training and the second for testing, while the third volume was excluded due to partial labeling. For the CREMI Synaptic Cleft benchmark, volume C was used for testing, while volumes A and B were used for training, as the original test data for this challenge are not publicly available. RETINA uses the sliding window method for inference (Cardoso et al., 2022), with adjacent regions of prediction partially overlapped to reduce missegmentation at the borders. Inference was conducted on Nvidia T4 GPUs on the Graham cluster of Digital Research Alliance of Canada.

Evaluation across all benchmark datasets is based on the following metrics:

Intersection over Union (IoU), defined as:

$$IoU = \frac{TP}{TP + FN + FP} \tag{S5}$$

where TP is the number of true positive predicted pixels, FN is the number of false negative predicted pixels, and FP is the number of false positive predicted pixels.

F-score, defined as:

$$F = \frac{2TP}{2TP + FP + FN} \tag{S6}$$

Precision, defined as:

$$Precision = \frac{TP}{TP + FP} \tag{S7}$$

Recall, defined as:

$$Recall = \frac{TP}{TP + FN} \tag{S8}$$

Mean False Distance (MFD), a Hausdorff-based metric that measures segmentation boundary errors, is defined as:

$$MFD = \frac{1}{|\Omega|} \sum_{x \in \Omega} d(x, \partial G) \tag{S9}$$

where Ω represents the set of misclassified pixels, $d(x, \partial G)$ denotes the shortest Euclidean distance from pixel x to the ground truth boundary ∂G, and $|\Omega|$ is the total number of misclassified pixels. These metrics collectively assess segmentation accuracy, boundary precision, and overall model performance across benchmark datasets.

Benchmark model implementation

Five models were selected for benchmarking: UNet-ResNet50 with randomly initialized parameters, UNet-ResNet50 pre-trained on CEM500K using MoCoV2, 2D TransUNet with randomly initialized parameters, 3D TransUNet with randomly initialized parameters, and 3D nnUNet. For UNet-ResNet50 with both random initialization and CEM500K pre-training, training procedures followed the details provided in (Conrad and Narayan, 2021). To ensure consistency, the pre-trained model was initialized with 200 epochs of MoCoV2 training on CEM500K, and the learned parameters were transferred to the fine-tuning model. During fine-tuning on benchmark training sets, the encoder was frozen. Augmentation functions and implementation parameters were adopted from the provided configuration files (`https://github.com/volume-em/cem-dataset`) (Conrad and Narayan, 2021). For 2D and 3D TransUNet with randomly initialized parameters, training and inference followed the same procedures. Augmentation functions and implementation details were based on the original TransUNet model (Chen et al., 2021, 2023). The nnUNet was employed using its 3D full resolution configuration, with hyperparameters determined through its self-configuration pipeline (Isensee et al., 2021, 2024). Checkpoints were saved at predefined iteration intervals for inference. Fine-tuning was conducted on Nvidia T4 GPUs within the Graham cluster. For inference, each benchmark used one of two Python scripts (`https://github.com/BaderLab/RETINA/tree/main/inference`): one for 2D models and another for 3D models. The two 3D models were applied to all datasets except the Perez dataset, as its images were randomly extracted from volume electron microscopy data as 2D slices rather than full 3D volumes. Finally, after generating predictions, metric scores were computed for evaluation.

REFERENCES

Buslaev, A., Iglovikov, V. I., Khvedchenya, E., Parinov, A., Druzhinin, M., and Kalinin, A. A. (2020). Albumentations: fast and flexible image augmentations. *Information* 11, 125

Cardoso, M. J., Li, W., Brown, R., Ma, N., Kerfoot, E., Wang, Y., et al. (2022). Monai: An open-source framework for deep learning in healthcare. *arXiv preprint arXiv:2211.02701*

Chen, J., Lu, Y., Yu, Q., Luo, X., Adeli, E., Wang, Y., et al. (2021). Transunet: Transformers make strong encoders for medical image segmentation. *arXiv preprint arXiv:2102.04306*

Chen, J., Mei, J., Li, X., Lu, Y., Yu, Q., Wei, Q., et al. (2023). 3d transunet: Advancing medical image segmentation through vision transformers. *arXiv preprint arXiv:2310.07781*

Conrad, R. and Narayan, K. (2021). Cem500k, a large-scale heterogeneous unlabeled cellular electron microscopy image dataset for deep learning. *Elife* 10, e65894

Dosovitskiy, A., Beyer, L., Kolesnikov, A., Weissenborn, D., Zhai, X., Unterthiner, T., et al. (2020). An image is worth 16x16 words: Transformers for image recognition at scale. *arXiv preprint arXiv:2010.11929*

Isensee, F., Jaeger, P. F., Kohl, S. A., Petersen, J., and Maier-Hein, K. H. (2021). nnu-net: a self-configuring method for deep learning-based biomedical image segmentation. *Nature methods* 18, 203–211

Isensee, F., Wald, T., Ulrich, C., Baumgartner, M., Roy, S., Maier-Hein, K., et al. (2024). nnu-net revisited: A call for rigorous validation in 3d medical image segmentation. In *International Conference on Medical Image Computing and Computer-Assisted Intervention* (Springer), 488–498

Perez, A. J., Seyedhosseini, M., Deerinck, T. J., Bushong, E. A., Panda, S., Tasdizen, T., et al. (2014). A workflow for the automatic segmentation of organelles in electron microscopy image stacks. *Frontiers in neuroanatomy* 8, 126
