# Supplementary material for: RETINA: Reconstruction-based pre-trained enhanced TransUNet for electron microscopy segmentation on the CEM500K dataset
Source: PLoS Comput Biol. 2025 May 28;21(5):e1013115. doi: 10.1371/journal.pcbi.1013115 (PMC12143494; doi:10.1371/journal.pcbi.1013115)
Supplement: S1 Table — The first column lists the function name. The second column shows the probability of each function being applied to the image. The third column indicates the augmentation set number to which each function belongs. Set 1 comprises the augmentation operations applied to both input images, ImageA and ImageB, as outlined in the RETINA pre-training methods section. Set 2 includes the operations applied exclusively to ImageA. The last column details the specific parameter settings for each function. (PDF) [file pcbi.1013115.s003.pdf]

**Table.** RETINA augmentation function settings. The first column lists the function name. The second column shows the probability of each function being applied to the image. The third column indicates the augmentation set number to which each function belongs. Set 1 comprises the augmentation operations applied to both input images, ImageA and ImageB, as outlined in the RETINA pre-training methods section. Set 2 includes the operations applied exclusively to ImageA. The last column details the specific parameter settings for each function.

| Augmentation | Probability | Set number | Parameters |
|---|---|---|---|
| HorizontalFlip | 0.5 | 1 | None |
| VeriticalFlip | 0.5 | 1 | None |
| Rotate | 0.5 | 1 | limit=90 |
| RandomResizedCrop | 1.0 | 1 | height=width=224<br>scale=(0.08, 1),<br>ratio=(0.5, 1.5) |
| RandomBrightnessContrast | 1.0 | 2 | brightness_limit=0.3<br>contrast_limit=0.3 |
| GaussNoise | 1.0 | 2 | var_limit=(400, 1200) |
| GaussianBlur | 1.0 | 2 | Default |
| CoarseDropout | 1.0 | 2 | max_holes=32<br>max_height=16<br>max_width=16<br>min_holes=1<br>fill_value=0<br>always_apply=False |
