# Supplementary material for: RETINA: Reconstruction-based pre-trained enhanced TransUNet for electron microscopy segmentation on the CEM500K dataset
Source: PLoS Comput Biol. 2025 May 28;21(5):e1013115. doi: 10.1371/journal.pcbi.1013115 (PMC12143494; doi:10.1371/journal.pcbi.1013115)
Supplement: S2 Table — (PDF) [file pcbi.1013115.s004.pdf]

**Table.** RETINA implementation parameters for pre-training.

| Config | Parameters |
|---|---|
| ResNet number of layers | (3, 4, 9) |
| ResNet width factor | 1 |
| ViT name | R50-VIT-B_16 |
| patch size | (16, 16) |
| hidden size | 192 |
| MLP dimension | 768 |
| number of heads | 12 |
| number of layers | 12 |
| attention dropout rate | 0.0 |
| transformer dropout rate | 0.1 |
| decoder channels | (256, 128, 64, 16) |
| skip channels | (512, 256, 64, 16) |
| number of skip | 3 |
| optimizer | Stochastic Gradient Descent |
| momentum | 0.9 |
| batch size | 256 |
| learning rate | 0.003 |
| weight decay | 0.0001 |
