# Supplementary material for: RETINA: Reconstruction-based pre-trained enhanced TransUNet for electron microscopy segmentation on the CEM500K dataset
Source: PLoS Comput Biol. 2025 May 28;21(5):e1013115. doi: 10.1371/journal.pcbi.1013115 (PMC12143494; doi:10.1371/journal.pcbi.1013115)
Supplement: S3 Table — (PDF) [file pcbi.1013115.s005.pdf]

Table. RETINA implementation parameters for fine-tuning.

Config	Parameters
ResNet number of layers	(3, 4, 9)
ResNet width factor	1
ViT name	R50-VIT-B_16
patch size	(16, 16)
hidden size	192
MLP dimension	768
number of heads	12
number of layers	12
attention dropout rate	0.0
transformer dropout rate	0.1
decoder channels	(256, 128, 64, 16)
skip channels	(512, 256, 64, 16)
number of skip	3
optimizer	Stochastic gradient descent
momentum	0.9
batch size	16
learning rate	0.003
weight decay	0.1
learning rate policy	Polynomial decay
