# Supplementary material for: RETINA: Reconstruction-based pre-trained enhanced TransUNet for electron microscopy segmentation on the CEM500K dataset
Source: PLoS Comput Biol. 2025 May 28;21(5):e1013115. doi: 10.1371/journal.pcbi.1013115 (PMC12143494; doi:10.1371/journal.pcbi.1013115)
Supplement: S4 Table — Abbreviations: ssTEM, serial section transmission electron microscopy; SBFSEM, serial block-face scanning electron microscopy; AT-SEM, array tomography scanning electron microscopy; FIBSEM, focused ion beam scanning electron microscopy. (PDF) [file pcbi.1013115.s006.pdf]

Table. Characteristics of the benchmark datasets. Abbreviations: ssTEM, serial section transmission electron microscopy; SBFSEM, serial block-face scanning electron microscopy; AT-SEM, array tomography scanning electron microscopy; FIBSEM, focused ion beam scanning electron microscopy. Characteristics of the benchmark datasets. Benchmark data can be accessed through the following links: CREMI Synaptic Clefts, Guay, Kasthuri++, Perez, UroCell.

**Benchmark**	**Microscopy type**	**Training set # of images**	**Training set image size**	**Testing set # of images**	**Testing set image size**	**Segmentation class(es)**
CREMI Synaptic Clefts	ssTEM	248	1250x1250	125	1250x1250	Synaptic Clefts
Guay	SBFSEM	49	800x800	23	800x800	Mitochondria<br>Canalicular channels<br>Alpha granules<br>Dense granules<br>Dense granule cores
Kasthuri++	AT-SEM	85	1463x1613	75	1334x1553	Mitochondria
Perez	SBEM	50	500x500	40	1000x1000	Mitochondria<br>Lysosomes<br>Nuclei<br>Nucleoli
UroCell	FIBSEM	3006	202x256	753	244x256	Mitochondria<br>Lysosomes
