# Supplementary material for: RETINA: Reconstruction-based pre-trained enhanced TransUNet for electron microscopy segmentation on the CEM500K dataset
Source: PLoS Comput Biol. 2025 May 28;21(5):e1013115. doi: 10.1371/journal.pcbi.1013115 (PMC12143494; doi:10.1371/journal.pcbi.1013115)
Supplement: S5 Table — Lysosomes, mitochondria, nuclei, and nucleoli within Perez benchmark are listed separately. Mean values of three independent runs are reported. (PDF) [file pcbi.1013115.s007.pdf]

Table. Comparison of segmentation F-score for RETINA versus benchmark models, including: randomly initialized (Rand. Init.) UNet-ResNet50, UNet-ResNet50 pre-trained on CEM500K, Rand. Init. 2D TransUNet, Rand. Init. 3D TransUNet and Rand. Init. nnUNet. Lysosomes, mitochondria, nuclei, and nucleoli within Perez benchmark are listed separately. Mean values of three independent runs are reported.

Benchmark	Training Iterations	Randomly Initialized UNet-ResNet50	CEM500K UNet-ResNet50	Rand. Init. 2D TransUNet	Rand. Init. 3D TransUNet	Rand. Init. nnUNet	RETINA
CREMI S.C.	5000	0.000	0.395	0.454	0.477	0.391	0.493
Guay	2500	0.623	0.637	0.420	0.661	0.662	0.711
Kasthuri++	10000	0.949	0.956	0.944	0.950	0.945	0.957
Perez	2500	0.919	0.950	0.943	–	–	0.959
Lysosomes	–	0.915	0.921	0.915	–	–	0.939
Mitochondria	–	0.914	0.941	0.917	–	–	0.941
Nuclei	–	0.992	0.995	0.995	–	–	0.996
Nucleoli	–	0.844	0.941	0.943	–	–	0.954
UroCell	1000	0.338	0.748	0.734	0.729	0.724	0.758
