# Supplementary material for: RETINA: Reconstruction-based pre-trained enhanced TransUNet for electron microscopy segmentation on the CEM500K dataset
Source: PLoS Comput Biol. 2025 May 28;21(5):e1013115. doi: 10.1371/journal.pcbi.1013115 (PMC12143494; doi:10.1371/journal.pcbi.1013115)
Supplement: S6 Table — Lysosomes, mitochondria, nuclei, and nucleoli within Perez benchmark are listed separately. Mean values of three independent runs are reported. (PDF) [file pcbi.1013115.s008.pdf]

Table. Comparison of segmentation Precision values for RETINA versus benchmark models, including: randomly initialized (Rand. Init.) UNet-ResNet50, UNet-ResNet50 pre-trained on CEM500K, Rand. Init. 2D TransUNet, Rand. Init. 3D TransUNet and Rand. Init. nnUNet. Lysosomes, mitochondria, nuclei, and nucleoli within Perez benchmark are listed separately. Mean values of three independent runs are reported.

Benchmark	Training Iterations	Rand. Init. UNet-ResNet50	CEM500K UNet-ResNet50	Rand. Init. 2D TransUNet	Rand. Init. 3D TransUNet	Rand. Init. nnUNet	RETINA
CREMI S.C.	5000	0.000	0.630	0.644	0.671	0.654	0.692
Guay	2500	0.648	0.613	0.517	0.640	0.639	0.722
Kasthuri++	10000	0.942	0.954	0.948	0.941	0.939	0.955
Perez	2500	0.919	0.937	0.944	–	–	0.952
Lysosomes	–	0.883	0.897	0.892	–	–	0.929
Mitochondria	–	0.882	0.938	0.900	–	–	0.944
Nuclei	–	0.994	0.994	0.993	–	–	0.995
Nucleoli	–	0.916	0.920	0.922	–	–	0.940
UroCell	1000	0.577	0.726	0.732	0.730	0.721	0.739
