# Supplementary material for: RETINA: Reconstruction-based pre-trained enhanced TransUNet for electron microscopy segmentation on the CEM500K dataset
Source: PLoS Comput Biol. 2025 May 28;21(5):e1013115. doi: 10.1371/journal.pcbi.1013115 (PMC12143494; doi:10.1371/journal.pcbi.1013115)
Supplement: S7 Table — Lysosomes, mitochondria, nuclei, and nucleoli within Perez benchmark are listed separately. Mean values of three independent runs are reported. (PDF) [file pcbi.1013115.s009.pdf]

**Table.** Comparison of segmentation Recall values for RETINA versus benchmark models, including: randomly initialized (Rand. Init.) UNet-ResNet50, UNet-ResNet50 pre-trained on CEM500K, Rand. Init. 2D TransUNet, Rand. Init. 3D TransUNet and Rand. Init. nnUNet. Lysosomes, mitochondria, nuclei, and nucleoli within Perez benchmark are listed separately. Mean values of three independent runs are reported.

| Benchmark | Training Iterations | Rand. Init. UNet-ResNet50 | CEM500K UNet-ResNet50 | Rand. Init. 2D TransUNet | Rand. Init. 3D TransUNet | Rand. Init. nnUNet | RETINA |
|---|---|---|---|---|---|---|---|
| CREMI S.C. | 5000 | 0.000 | 0.287 | 0.351 | 0.370 | 0.279 | 0.383 |
| Guay | 1000 | 0.599 | 0.664 | 0.354 | 0.682 | 0.687 | 0.699 |
| Kasthuri++ | 10000 | 0.957 | 0.958 | 0.940 | 0.959 | 0.954 | 0.959 |
| Perez | 2500 | 0.919 | 0.962 | 0.959 | – | – | 0.963 |
| Lysosomes | – | 0.952 | 0.947 | 0.941 | – | – | 0.950 |
| Mitochondria | – | 0.948 | 0.942 | 0.935 | – | – | 0.940 |
| Nuclei | – | 0.990 | 0.996 | 0.996 | – | – | 0.996 |
| Nucleoli | – | 0.784 | 0.963 | 0.964 | – | – | 0.966 |
| UroCell | 1000 | 0.239 | 0.773 | 0.734 | 0.727 | 0.726 | 0.777 |
