# Supplementary material for: RETINA: Reconstruction-based pre-trained enhanced TransUNet for electron microscopy segmentation on the CEM500K dataset
Source: PLoS Comput Biol. 2025 May 28;21(5):e1013115. doi: 10.1371/journal.pcbi.1013115 (PMC12143494; doi:10.1371/journal.pcbi.1013115)
Supplement: S8 Table — The Mean False Distance is based on the mean of the bidirectional directed Hausdorff Distance between the ground truth and the predicted segmentation. Lysosomes, mitochondria, nuclei, and nucleoli within Perez benchmark are listed separately. Mean values of three independent runs are reported. (PDF) [file pcbi.1013115.s010.pdf]

Table. Comparison of segmentation Mean False Distance in pixel/voxel distance for RETINA versus benchmark models, including: randomly initialized (Rand. Init.) UNet-ResNet50, UNet-ResNet50 pre-trained on CEM500K, Rand. Init. 2D TransUNet, Rand. Init. 3D TransUNet and Rand. Init. nnUNet. The Mean False Distance is based on the mean of the bidirectional directed Hausdorff Distance between the ground truth and the predicted segmentation. Lysosomes, mitochondria, nuclei, and nucleoli within Perez benchmark are listed separately. Mean values of three independent runs are reported.

Benchmark	Training Iterations	Rand. Init. UNet-ResNet50	CEM500K UNet-ResNet50	Rand. Init. 2D TransUNet	Rand. Init. 3D TransUNet	Rand. Init. nnUNet	RETINA
CREMI S.C.	5000	NaN	121.30	116.12	111.33	135.91	107.77
Guay	1000	133.66	138.68	191.21	136.04	134.18	123.21
Kasthuri++	10000	134.35	108.87	133.64	127.54	112.05	102.70
Perez	2500	34.07	24.98	23.39	–	–	20.03
Lysosomes	–	42.90	39.64	25.07	–	–	21.93
Mitochondria	–	43.98	22.70	31.93	–	–	24.01
Nuclei	–	22.54	18.83	19.19	–	–	18.12
Nucleoli	–	26.85	18.73	17.35	–	–	16.06
UroCell	1000	88.67	59.44	56.51	52.74	53.52	51.67
