# Supplementary material for: RETINA: Reconstruction-based pre-trained enhanced TransUNet for electron microscopy segmentation on the CEM500K dataset
Source: PLoS Comput Biol. 2025 May 28;21(5):e1013115. doi: 10.1371/journal.pcbi.1013115 (PMC12143494; doi:10.1371/journal.pcbi.1013115)
Supplement: S9 Table — Metrics include the CREMI score (mean of ADGT and ADF), false positives (FP), false negatives (FN), 1 - F-score, ADGT (average distance of any predicted cleft voxel to the closest ground truth cleft voxel), and ADF (average distance of any ground truth cleft voxel to the closest predicted cleft voxel). Lower values indicate better performance. Models were trained on volumes A and B and evaluated on volume C. The performance of the randomly initialized UNet-ResNet50 could not be calculated due to its low accuracy. (PDF) [file pcbi.1013115.s011.pdf]

Table. Performance on the CREMI dataset using the same evaluation metrics as the challenge. Metrics include the CREMI score (mean of ADGT and ADF), false positives (FP), false negatives (FN), 1 - F-score, ADGT (average distance of any predicted cleft voxel to the closest ground truth cleft voxel), and ADF (average distance of any ground truth cleft voxel to the closest predicted cleft voxel). Lower values indicate better performance. Models were trained on volumes A and B and evaluated on volume C. The performance of the randomly initialized UNet-ResNet50 could not be calculated due to its low accuracy.

Model	CREMI score	FP	FN	1 - F-score	ADGT	ADF
Rand. Init. UNet-ResNet50	–	–	–	–	–	–
CEM500K UNet-ResNet50	34.01	40490	29789	0.055	39.36	28.66
Rand. Init. 2D TransUNet	35.26	21832	47534	0.069	23.83	46.68
Rand. Init. 3D TransUNet	32.11	19807	45815	0.063	22.13	42.09
Rand. Init. nnUNet	43.08	35975	52854	0.091	32.98	53.17
RETINA	32.25	23465	46393	0.046	30.01	34.49
