# Supplementary material for: RETINA: Reconstruction-based pre-trained enhanced TransUNet for electron microscopy segmentation on the CEM500K dataset
Source: PLoS Comput Biol. 2025 May 28;21(5):e1013115. doi: 10.1371/journal.pcbi.1013115 (PMC12143494; doi:10.1371/journal.pcbi.1013115)
Supplement: S10 Table — The models include randomly initialized (Rand. Init.) 2D TransUNet, 2D TransUNet pre-trained on ImageNet, 2D TransUNet using ImageNet pre-trained parameters followed by pre-training on CEM500K, and RETINA. These models were fine-tuned and evaluated on all benchmark datasets listed in the first column. The IoU scores represent the best performance achieved with the specific number of training iterations shown in the second column. Lysosomes, mitochondria, nuclei, and nucleoli within Perez benchmark are listed separately. Mean values of three independent runs are reported. (PDF) [file pcbi.1013115.s012.pdf]

Table. Comparison of IoU scores for models pre-trained on different datasets. The models include randomly initialized (Rand. Init.) 2D TransUNet, 2D TransUNet pre-trained on ImageNet, 2D TransUNet using ImageNet pre-trained parameters followed by pre-training on CEM500K, and RETINA. These models were fine-tuned and evaluated on all benchmark datasets listed in the first column. The IoU scores represent the best performance achieved with the specific number of training iterations shown in the second column. Lysosomes, mitochondria, nuclei, and nucleoli within Perez benchmark are listed separately. Mean values of three independent runs are reported.

**Benchmark**	**Training Iterations**	**Rand. Init. 2D TransUNet**	**ImageNet 2D TransUNet**	**ImageNet & CEM500K 2D TransUNet**	**RETINA**
CREMI S.C.	5000	0.294	0.314	0.305	**0.327**
Guay	1000	0.223	0.390	0.359	**0.513**
Kasthuri++	10000	0.894	0.910	0.914	**0.916**
Perez	2500	0.893	0.909	0.914	**0.919**
Lysosomes	–	0.845	0.844	0.862	**0.885**
Mitochondria	–	0.847	0.889	**0.892**	0.890
Nuclei	–	0.990	0.990	0.991	**0.991**
Nucleoli	–	0.891	**0.914**	0.910	0.910
UroCell	1000	0.579	**0.649**	0.596	0.610
