# Supplementary material for: RETINA: Reconstruction-based pre-trained enhanced TransUNet for electron microscopy segmentation on the CEM500K dataset
Source: PLoS Comput Biol. 2025 May 28;21(5):e1013115. doi: 10.1371/journal.pcbi.1013115 (PMC12143494; doi:10.1371/journal.pcbi.1013115)
Supplement: S11 Table — The values represent the mean time per iteration, averaged over the entire training process. Rand. Init., randomly initialized. (PDF) [file pcbi.1013115.s013.pdf]

Table. Average training time per iteration for each model on each dataset, measured using Nvidia A100 GPUs on the Narval cluster of the Digital Research Alliance of Canada. The values represent the mean time per iteration, averaged over the entire training process. Rand. Init., randomly initialized.

Dataset	Model	Time of each iteration (second)
Cremi	Rand. Init. UNet-ResNet50	0.12
	CEM500K UNet-ResNet50	0.12
	Rand. Init. 2D TransUNet	0.14
	Rand. Init. 3D TransUNet	0.15
	Rand. Init. nnUNet	0.13
	RETINA	0.13
UroCell	Rand. Init. UNet-ResNet50	0.10
	CEM500K UNet-ResNet50	0.10
	Rand. Init. 2D TransUNet	0.12
	Rand. Init. 3D TransUNet	0.16
	Rand. Init. nnUNet	0.13
	RETINA	0.12
Guay	Rand. Init.	0.13
	CEM500K UNet-ResNet50	0.13
	Rand. Init. 2D TransUNet	0.14
	Rand. Init. 3D TransUNet	0.17
	Rand. Init. nnUNet	0.14
	RETINA	0.14
Kasthuri	Rand. Init. UNet-ResNet50	0.12
	CEM500K UNet-ResNet50	0.12
	Rand. Init. 2D TransUNet	0.12
	Rand. Init. 3D TransUNet	0.15
	Rand. Init. nnUNet	0.13
	RETINA	0.12
Perez	Rand. Init. UNet-ResNet50	0.10
	CEM500K UNet-ResNet50	0.10
	Rand. Init. 2D TransUNet	0.12
	Rand. Init. 3D TransUNet	–
	Rand. Init. nnUNet	–
	RETINA	0.12
