# Supplementary material for: RETINA: Reconstruction-based pre-trained enhanced TransUNet for electron microscopy segmentation on the CEM500K dataset
Source: PLoS Comput Biol. 2025 May 28;21(5):e1013115. doi: 10.1371/journal.pcbi.1013115 (PMC12143494; doi:10.1371/journal.pcbi.1013115)
Supplement: Attachment — Submitted filename: response_to_reviewer.pdf [file pcbi.1013115.s014.pdf]

Response made to manuscript Paper PCOMPBIOL-D-24-01938

The authors sincerely thank the editor and reviewers for their insightful comments and constructive suggestions, which have significantly contributed to improving the quality of this manuscript. We have revised the manuscript in accordance with the provided feedback.
Below is a summary of the major revisions:

- Two 3D benchmark models have been added, and their results are now included, with relevant figures updated accordingly.
- A discussion has been added to propose the idea of extending RETINA into 3D as a future direction.
- Four additional evaluation metrics have been incorporated, and all benchmark datasets and models have been reevaluated accordingly.
- The experiments on model iteration time have been supplemented, and the results are now included in the revised manuscript.
- The presented number of iteration, model names and ordering have been standardized for consistency across tables, figures, and the main text.

Please find below our detailed responses to each comment.

Response to the reviewers

Reviewer 1

Reviewer Comment 1.1
Xing et al. describe RETINA, a machine-learning method for segmenting ultrastructural features in electron microscopy (EM) images. Segmenting ultrastructures such as mitochondria, lysosomes, nucleoli, etc. in EM datasets is critical for their analysis since manual segmentation does not scale to large datasets. The main contribution of the authors is to implement pretraining on a related dataset into their model training to improve performance. Pretraining can leverage large amounts of unlabeled data avoiding higher costs for label generation. The authors show that pretraining improves model performance in comparison to a baseline that does not use pretraining, as well as another baseline.

The paper is written clearly, and the data is presented well. However, there are several shortcomings in the model chosen for the benchmark datasets, experiments performed and presented, the comparison models, and the metrics used.

Response: We kindly thank the reviewer for their comments and have addressed the shortcomings point-by-point below.

Reviewer Comment 1.2
The authors benchmark their model on several datasets that are 3d, in that they extend in all 3d

dimensions. It has been long established that leveraging this 3d context improves performance, yet, the authors have chosen a model that uses 2d convolutions. This part is not entirely clear though, because the model is not fully presented in the paper; I referred to the original manuscript in which the model architecture of Transunet was presented (Chen et al., 2021).

Response: Thank you for your insightful comment. We apologize for not clearly explaining the rationale behind our choice of a 2D architecture.

The main reason that we use 2D convolutions is to ensure compatibility with the pre-training framework. Since the CEM500K dataset consists entirely of 2D images, a pre-training architecture based on 2D convolutional networks is necessary to align with the image dimensions. This 2D design is also inspired from the approach established in the CEM500K study [4], which demonstrated that pre-training on this dataset enhances the performance of 2D models due to information-rich and non-redundant properties of CEM500K. We have now clarified this reasoning in the "Model Architecture: RETINA Pre-training" section in our manuscript. We did incorporate 3D information in the case of the UroCell training data, which is an isotropic volume (where the x, y, and z dimensions have equal resolution). We trained the model on a combination of axial (xy), sagittal (yz), and coronal (xz) slices. During inference, we used an orthoplane prediction strategy, generating predictions on all three planes and averaging their output scores to improve accuracy. This has been shown to improve performance, in agreement with the reviewer's statement about use of 3D data [3]. We have now added this explanation to the "Results: Evaluation of RETINA on Benchmark Data" section and the supplementary material under "Implementation: Inference Methods."

We agree that using 3D information is a promising avenue of research to explore, thus we propose extending RETINA to a fully 3D architecture in future work. One potential approach involves generating and curating a sufficiently large 3D EM dataset—leveraging advancements in volumetric electron microscopy, such as the datasets available in OpenOrganelle [9]—to pre-train a robust 3D encoder. We have added a discussion of this point to the "Discussion" section.

To address the reviewer's point that the TransUNet model was not fully presented, we have revised the sections "Model Architecture: RETINA Pre-training" and "Model Architecture: Fine-tuning Using TransUNet" to provide a more detailed description of the TransUNet architecture, explain how RETINA was designed based on it, and clarify the compatibility between the CEM500K dataset, the pre-training framework, and the fine-tuning process through a 2D-based design. Additionally, we have expanded the description of the RETINA model structure in Supplementary Material Section 1.4, "RETINA Pre-training Methods."

Reviewer Comment 1.3

The baseline the authors compare their model to appear to be 2d models as well which do not represent the state of the art. Just as one point of reference, the CREMI leaderboard (`https://cremi.org/leaderboard/`) lists multiple methods applied for synapse detection. CleftNet, one of the high-scoring methods, uses a 3d residual UNet (`https://arxiv.org/pdf/2101.04266`). This is just one example for a stronger baseline the authors could have considered.

Response: We acknowledge that our initial comparison did not comprehensively include 3D benchmark models. To address this, we conducted a thorough review of the CREMI competition and its top-performing models on the leaderboard. While we attempted to reimplement CleftNet, we were unable to do so due to the lack of publicly available code in its GitHub repository (`https://github.com/divelab/cleftnet`). Instead, we identified nnUNet as a strong benchmark model for

several reasons. First, even its earlier version, which lacked residual structures in the encoder, ranked as one of the top models on the CREMI leaderboard for synaptic cleft detection task based on the F-score [11, 12]. The latest nnUNet model incorporates a 3D residual UNet architecture, which has been reported to achieve improved performance (`https://github.com/MIC-DKFZ/nnUNet` in "documentation/how_to_use_nnunet.md"). Second, nnUNet has demonstrated strong generalization across diverse datasets beyond the CREMI challenge, making it a reliable benchmark model. Third, its well-documented and publicly available implementation supports a correct and fair comparison. Therefore, we have incorporated nnUNet (3D full-resolution variant with a residual encoder) as one of our additional benchmark models. We also included 3D TransUNet [2] as another 3D baseline. This model is an extension of 2D TransUNet to 3D images and has demonstrated strong performance in 3D medical image segmentation tasks. Originally, we benchmarked only against the 2D TransUNet to highlight the impact of RETINA's pre-training strategy, given that RETINA is built upon the 2D TransUNet architecture. However, inspired by the reviewer's point, we realized it is also important to benchmark the 3D version of TransUNet for a more comprehensive comparison.

Both newly added 3D models (nnUNet and 3D TransUNet) do not have publicly available pre-trained versions. Thus, we trained them from scratch with the randomly initialized weight and the training was under the same conditions as other models. Specifically, we adopted identical training and testing splits, the same number of training iterations, and consistent evaluation metrics as other benchmarks. Model hyperparameters follow the default settings reported in the original publications. Further implementation details are provided in the "Benchmark Model Implementation" subsection of the Supplementary Materials.

After benchmarking, RETINA continues to outperform both nnUNet and 3D TransUNet across all evaluation metrics, including the additional metrics introduced in the revision. We attribute this performance gain to RETINA effective pre-training on the CEM500K dataset.

All evaluations related to the 3D benchmarks have been updated in the "Results" section of the main text. Additionally, the "Implementation: Benchmark Model Implementation" section in the supplementary materials has been revised accordingly. The updates are also reflected in Figure 3, Figure 4, and Table 1. And Figure S1 was added.

Reviewer Comment 1.4

The authors chose to use IoU for all their benchmarks. This is a limited metrics because it does not treat voxels that separate individual objects differently from those that are merely at the edge. Yet, merging two distinct objects has a much higher impact on downstream analyses. For instance, the CREMI challenge uses an object based metric (`https://cremi.org/metrics/#clefts`). A side effect of only using IoU is that the presented performance cannot be compared with the performances published on the cremi challenge. If a challenge like CREMI publishes metrics, they should be used.

Still, the pretraining as implemented by the authors improves performance according to one metric (when compared to its not-pretrained self). To make the impact clear however, more evaluations and metrics should have been presented. For instance, it is not clear if the performance gap outweighs the additional cost of pretraining, etc, etc.

Response: This is a good point. We reviewed several EM segmentation studies [9, 16] and identified additional metrics—F-score, Precision, Recall, and Mean False Distance (MFD)—that are well-suited for our semantic segmentation task. We have incorporated these metrics into our benchmark evaluations, and the results are now presented in the supplementary material tables (From Table S5 to Table S8).

Second, the CREMI challenge applies a threshold-based tolerance in false positive and false negative calculations. Specifically, voxels labeled as synaptic clefts that fall beyond a threshold distance of 200 from the ground truth are considered false positives, while ground truth synaptic cleft voxels beyond the same threshold from predicted labels are considered false negatives. This approach better reflects synaptic cleft detection performance. To ensure alignment with the challenge, we also evaluated CREMI predictions using the exact metrics applied in their second task, including FP, FN, 1 - F-score, ADGT, and ADF (Table S9). RETINA achieves the best performance on these metrics among all models. The "Results: Evaluation of RETINA on Benchmark Data" section in the main text has also been updated to include performance assessments based on these metrics. Correspondingly, metric definitions have been added to the supplementary material under "Implementation: Inference Methods." RETINA consistently achieves higher scores across all added metrics compared to benchmark models. The corresponding description has been added to the "Results: RETINA Achieves the Best Segmentation Accuracy on EM Benchmarks" section.

Additionally, inspired by the reviewer's comment, we realized that our segmentation task was not clearly defined. Our study focuses on semantic segmentation, and all selected benchmark models are designed for this task, with ground truth labels in semantic format. Semantic segmentation assigns a class identifier (ID) to each pixel/voxel, whereas instance segmentation differentiates individual objects with unique IDs [4, 5, 10, 13].

Since RETINA is primarily designed for semantic segmentation, being built upon the TransUNet architecture used for semantic segmentation, we focus exclusively on the task of semantic segmentation. Accordingly, only semantic segmentation benchmarks and evaluation metrics were considered. Applying RETINA to instance segmentation, which requires distinguishing individual objects with unique IDs, would necessitate additional post-processing steps (e.g., the Watershed algorithm [15]) and extensive hyperparameter tuning. These extra steps introduce variables that could confound the evaluation of the model's core performance. For these reasons, we limited our experiments to end-to-end semantic segmentation tasks, emphasizing the effects of pre-training and architectural design. We have clarified the focus on semantic segmentation in the "Introduction" and "Model Architecture" sections of the main text.

Nonetheless, RETINA can be adapted for instance segmentation with appropriate post-processing applied to its semantic predictions. We added the adaptability of our approach to instance segmentation in the future directions section of the "Discussion".

Regarding the issue of merging two objects, we acknowledge that using only IoU is not sufficient. Since we are not using instance based segmentation, we can't evaluate this aspect. We have mentioned this in the discussion.

Reviewer Comment 1.5

Overall, the impact of this work on the science that is using segmentations like the once created by RETINA is not made clear enough. The introduction claims that "deep learning-based segmentation has shown promise, its accuracy to automatically segment cellular structures in EM data remains insufficient compared to expert manual results." There are many publications to list here that show that this is not true. Many automated methods now going back almost 10 years have shown performances similar to human annotators for ultrastructural segmentation. That is not to say that there are no improvements to be had, especially when it comes to efficiency along the lines of what RETINA tries to achieve.

Response: We apologize for the inappropriate claim and appreciate the reviewer's insight. We agree

that deep learning-based segmentation methods can reach expert-level performance on specific tasks, as shown by prior work [1, 6, 14, 17]. We revised to claim that while many deep learning-based methods have demonstrated expert-level performance for specific segmentation tasks [1, 6, 14, 17], deep learning models often lack generalizable segmentation performance across diverse EM datasets due to the inherent variability and complexity of nanoscale EM structures. Consequently, extensive manual annotation and task-specific fine-tuning remain necessary to adapt existing models to new datasets [7, 8, 16], indicating that developing an effective and efficient automated EM segmentation method remains an open challenge.

Our work with RETINA presents a deep learning-based segmentation method designed to provide effective and efficient segmentation performance across diverse EM datasets.

We have revised the "Abstract" and "Introduction" sections of the main text.

Reviewer 2

Reviewer Comment 2.1

Xing et al. presented a hybrid approach, RETINA, for EM segmentation. By combining the widely used TransUNet backbone with pre-trained features from the CEM500K dataset, the authors suggested that the hybrid method outperformed the other approaches in terms of speed and accuracy. It is a timely work given the rapid expansion of volume EM datasets in recent years. However, I recommend addressing the overall accuracy of the manuscript, particularly in the following areas:

Response: We kindly thank the reviewer for their comments and have addressed them point-by-point below.

Reviewer Comment 2.2

1. Could you provide the iteration time for each model? This information is essential for accurately estimating the computational resources required to achieve a certain IoU.

Response: Thank you for the comment. We have added a table documenting the iteration time for each model, which is now available in the supplementary material (Table S11). This table presents the average training time per iteration for each model across different datasets, including the newly added benchmark models. The results indicate that computational resource requirements are comparable across models.

The inclusion of iteration time data provides insights into the computational resources needed for each model to achieve a given performance level, as measured by various metrics. Based on this analysis, we have revised the main text and updated the "Discussion" section as follows: "Since times per iteration are nearly identical across models, RETINA's faster convergence indicates that it requires fewer iterations to achieve high performance, reducing overall training time (Table S11)."

Reviewer Comment 2.3

2. Discrepancies in the numbers reported in Table 1, Table S5, and Figure 3. For example, in line 145, it is stated that "The UroCell benchmark demonstrates the most significant IoU increase, over 150%". I was not able to derive this number from Table 1. The Guay numbers in Table 1 do not match those in Table S5 and Figure 3. The UroCell Random Init. value of 0.203 in Table 1 appears

to be incorrect. Iteration numbers of Gray, Perez, and Kasthuri++ in Figure 3 do not match with those in Table 1. The spelling of "Kathuri" in Figure 3 should be "Kasthuri".

Response: We apologize for these discrepancies and have revised all the inconsistencies mentioned by the reviewer. First, we initially used percentage representation to illustrate RETINA's performance improvement over benchmark models, following prior work [4]. The improvement of over 150% was calculated as (IoU of RETINA - IoU of the randomly initialized UNet-ResNet50) / IoU of the randomly initialized UNet-ResNet50. However, we recognize that this expression may cause confusion. To avoid ambiguity, we have replaced all related descriptions with direct metric values. For example, the revised statement now reads: "The UroCell benchmark shows that RETINA achieves an IoU of 0.610, the highest among all models." The detailed revisions are reflected in the "Results: RETINA Achieves the Best Segmentation Accuracy on EM Benchmarks" section.

Second, the previously reported iteration number for Guay in Table 1 was 1000, following the iteration number used in the prior work we are comparing ourselves to [4]. However, through further experimentation, we found that RETINA had not fully converged at 1000 iterations. To ensure a fair comparison, we extended the experiment to 2500 iterations, where convergence was observed. We apologize that the discrepancy in iteration counts may have caused confusion. We have now revised Table 1 to report the Guay dataset performance at 2500 iterations. Its performance at 1000 iterations can still be found in Figure 3.

Third, regarding the UroCell "Random Init." value (0.203) in Table 1, we think the reviewer raised this point due to a comparison with Table S5 (now Table S10 in the revised version), where a different "Random Init." value (0.579) was listed. This discrepancy was caused by a column name misinterpretation—"Random Init." in Table S5 (Table S10 in the revised version) referred to the randomly initialized 2D TransUNet model, not the randomly initialized UNet-ResNet50 model. We apologize for this issue. To clarify this, we have renamed the column in Table S5 (Table S10 in the revised version) as "Rand. Init. 2D TransUNet" and added an explanatory note in the table caption. Furthermore, we have double-checked the UroCell IoU value for the randomly initialized UNet-ResNet50 model, confirming it as 0.203, which aligns with the published benchmark result (0.208, shown in its Table 1) [4], reaffirming that the randomly initialized UNet-ResNet50 model performs weakly on UroCell.

Fourth, to clearly illustrate the convergence trend, we plotted only a subset of iterations that captures the most significant increase in IoU, ensuring better visualization of the progression toward convergence. For example, models trained on the Kasthuri++ dataset reach convergence at approximately 1000 iterations, making additional iterations unnecessary for effectively depicting the learning process. The final reported performance in Table 1 reflects the performance evaluated at the final total number of iterations that is aligned with the selected iteration numbers in the published CEM500K study that we compare to [4]. We have now included an explanation in the caption of Figure 3 for clarity.

Finally, "Kathuri" has been corrected to "Kasthuri" throughout the manuscript.

Reviewer Comment 2.4

3. The names of benchmarks in Table 1 should be better defined to ensure consistency with the main text. For example, "CEM500K moco" in Table 1 should be explicitly linked to "UNet-ResNet50 model pre-trained with MoCoV2" in line 126 and the "MoCoV2 pre-trained model" in line 158. "Random Init." should be clearly defined as the "randomly initialized UNet-ResNet50 model" in line 131. The "non-pre-trained UNet-ResNet50 model" in line 172 is unclear and requires clarification.

Response: Thank you for the comment. We have revised and standardized the naming format for models as "randomly initialized / pre-training dataset + model architecture name." Specifically, "CEM500K MoCo" in Table 1 has been updated to "CEM500K UNet-ResNet50." Corresponding name changes have also been applied throughout the text, as reflected in the "Results: Evaluation of RETINA on Benchmark Data" section. Additionally, "Random Init." has been replaced with "Randomly Initialized UNet-ResNet50" in the text, figures, and tables.

Furthermore, we have replaced the term "non-pre-trained" with "randomly initialized" to maintain consistency across the manuscript.

Reviewer Comment 2.5

4. It would be better to have the order of Benchmarks be consistent between Figure 3 and Figure S5.

Response: We have adjusted the order to ensure consistency between Figure 3 and Table S5 (now Table S10 in the revised version). (We assume the reviewer was referring to Table S5, as Figure S5 was not included in the original submission).

References

[1] Boaro, A., Kaczmarzyk, J. R., Kavouridis, V. K., Harary, M., Mammi, M., Dawood, H., et al. (2022). Deep neural networks allow expert-level brain meningioma segmentation and present potential for improvement of clinical practice. *Scientific Reports* 12, 15462

[2] Chen, J., Mei, J., Li, X., Lu, Y., Yu, Q., Wei, Q., et al. (2023). 3d transunet: Advancing medical image segmentation through vision transformers. *arXiv preprint arXiv:2310.07781*

[3] Conrad, R., Lee, H., and Narayan, K. (2020). Enforcing prediction consistency across orthogonal planes significantly improves segmentation of fib-sem image volumes by 2d neural networks. *Microscopy and Microanalysis* 26, 2128–2130

[4] Conrad, R. and Narayan, K. (2021). Cem500k, a large-scale heterogeneous unlabeled cellular electron microscopy image dataset for deep learning. *Elife* 10, e65894

[5] Funke, J., Tschopp, F., Grisaitis, W., Sheridan, A., Singh, C., Saalfeld, S., et al. (2018). Large scale image segmentation with structured loss based deep learning for connectome reconstruction. *IEEE transactions on pattern analysis and machine intelligence* 41, 1669–1680

[6] Greenwald, N. F., Miller, G., Moen, E., Kong, A., Kagel, A., Dougherty, T., et al. (2022). Whole-cell segmentation of tissue images with human-level performance using large-scale data annotation and deep learning. *Nature biotechnology* 40, 555–565

[7] Guay, M. D., Emam, Z. A., Anderson, A. B., Aronova, M. A., Pokrovskaya, I. D., Storrie, B., et al. (2021). Dense cellular segmentation for em using 2d–3d neural network ensembles. *Scientific reports* 11, 2561

[8] Han, M., Bushong, E. A., Segawa, M., Tiard, A., Wong, A., Brady, M. R., et al. (2023). Spatial mapping of mitochondrial networks and bioenergetics in lung cancer. *Nature* 615, 712–719

[9] Heinrich, L., Bennett, D., Ackerman, D., Park, W., Bogovic, J., Eckstein, N., et al. (2021). Whole-cell organelle segmentation in volume electron microscopy. *Nature* 599, 141–146

[10] Heinrich, L., Funke, J., Pape, C., Nunez-Iglesias, J., Saalfeld, S., Schnabel, J., et al. (2018). Synaptic cleft segmentation in non-isotropic volume electron microscopy of the complete. *Drosophila brain*

[11] Isensee, F., Jaeger, P. F., Kohl, S. A., Petersen, J., and Maier-Hein, K. H. (2021). nnu-net: a self-configuring method for deep learning-based biomedical image segmentation. *Nature methods* 18, 203–211

[12] Isensee, F., Wald, T., Ulrich, C., Baumgartner, M., Roy, S., Maier-Hein, K., et al. (2024). nnu-net revisited: A call for rigorous validation in 3d medical image segmentation. In *International Conference on Medical Image Computing and Computer-Assisted Intervention* (Springer), 488–498

[13] Januszewski, M., Kornfeld, J., Li, P. H., Pope, A., Blakely, T., Lindsey, L., et al. (2018). High-precision automated reconstruction of neurons with flood-filling networks. *Nature methods* 15, 605–610

[14] Khadangi, A., Boudier, T., and Rajagopal, V. (2021). Em-stellar: benchmarking deep learning for electron microscopy image segmentation. *Bioinformatics* 37, 97–106

[15] Kornilov, A., Safonov, I., and Yakimchuk, I. (2022). A review of watershed implementations for segmentation of volumetric images. *Journal of Imaging* 8, 127

[16] Parlakgül, G., Arruda, A. P., Pang, S., Cagampan, E., Min, N., Güney, E., et al. (2022). Regulation of liver subcellular architecture controls metabolic homeostasis. *Nature* 603, 736–742

[17] Zeng, T., Wu, B., and Ji, S. (2017). Deepem3d: approaching human-level performance on 3d anisotropic em image segmentation. *Bioinformatics* 33, 2555–2562
